# Spin-photon module for scalable network architecture in quantum dots

**DOI:** 10.1038/s41598-020-61976-2

**Published:** 2020-03-19

**Authors:** Xing-Yu Zhu, Tao Tu, Ao-Lin Guo, Zong-Quan Zhou, Guang-Can Guo, Chuan-Feng Li

**Affiliations:** 1 Key Laboratory of Quantum Information, University of Science and Technology of China, Chinese Academy of Sciences, Hefei, 230026 P.R. China; 2 Department of Physics and Astronomy, University of California at Los Angeles, California, 90095 USA

**Keywords:** Qubits, Quantum optics

## Abstract

Reliable information transmission between spatially separated nodes is fundamental to a network architecture for scalable quantum technology. Spin qubit in semiconductor quantum dots is a promising candidate for quantum information processing. However, there remains a challenge to design a practical path from the existing experiments to scalable quantum processor. Here we propose a module consisting of spin singlet-triplet qubits and single microwave photons. We show a high degree of control over interactions between the spin qubit and the quantum light field can be achieved. Furthermore, we propose preparation of a shaped single photons with an efficiency of 98%, and deterministic quantum state transfer and entanglement generation between remote nodes with a high fidelity of 90%. This spin-photon module has met the threshold of particular designed error-correction protocols, thus provides a feasible approach towards scalable quantum network architecture.

## Introduction

To develop an architecture is one of the most important challenges in the field of quantum computation and quantum communication. The design of architecture is to find a realistic path from the feasible state-of-art technology to scalable quantum information processing. Plenty of progress has been made on a monolithic architecture, while for many physical platforms a network architecture may be more achievable^1–6^. Deterministic and efficient quantum state transfer between spatially separated qubits is an essential part of large-scale network architecture^7,8^. This scheme requires a universal module that is capable of sending, receiving, storing and processing quantum information encoded in temporal shaped single photons. In recent years, this kind of module has been proposed and demonstrated in several physical systems such as trapped ions^9,10^, single atoms^11^, and superconducting circuits^12–14^. However these experiments remains in the stage of proof-of-principle, there is an ongoing effort to find alternative platforms that do not require fine-tuning of parameters and can be easily scaled to large numbers of qubits.

The recent significant advance of electron spin qubits in semiconductor quantum dots provides an attractive candidate to realize the building blocks of the network architecture^15–18^. In this context, spin qubits in quantum dots features various desirable properties. This system takes advantage of long coherence times associated with spin states and high fidelity manipulations have been established^19–23^. Furthermore this system is compatible with industrial semiconductor technology. It is timely to seek a network approach for spin qubits in quantum dots. However, this scheme is more challenging to realize, because of the difficulty in achieving reliable spin-photon interface. One way to remedy these issues is to utilize a spin qubit with chargelike states. ref. ^24^ proposed a strong coupling mechanism between a spin singlet-triplet qubit with a resonator, which made an initial and important step towards the network architecture.

**Figure 1:**
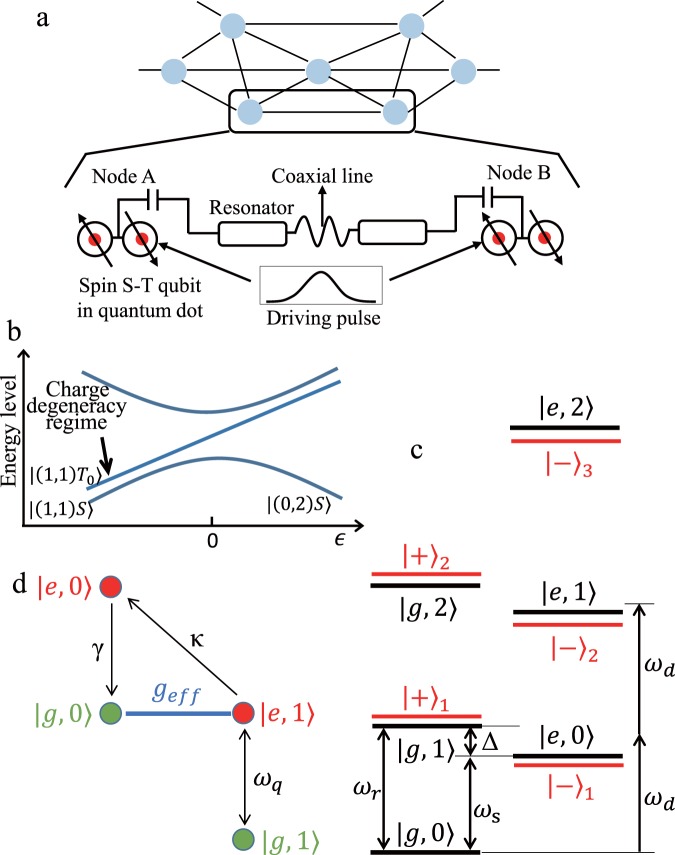
Spin-photon modules for a network architecture. (**a**) In the envisaged network, many spin-photon modules are connected by coaxial lines. Each node consists of a spin single-triplet qubit in double quantum dots coupled to a superconducting resonator. The driving pulses are applied on the gate electrodes of the quantum dots to generate the shaped single photons in the resonator. (**b**) The energy level diagram of the spin single-triplet qubit. (**c**) The energy spectrum of the spin-photon hybrid system. (**d**) Illustration of the key process: an effective coupling *g*_*eff*_ between the states $\left|g0\right\rangle$ and $\left|e1\right\rangle$. *κ* is the photon decay of the resonator and *γ* is the decoherence of the spin qubit.

Here we describe a scheme for the network architecture based on a spin-photon module comprising single microwave photons in resonator coupled to spin singlet-triplet qubits in quantum dots. Based on ref. ^24^, a driving microwave pulse is applied to the module and mediates tunable interactions between the electron spins and the photons. The single photons with temporal shape can be generated by changing the amplitude and phase of the driving pulse over time. Thus the spin qubits are entangled to photons, which constitute communication carriers enabling the state transfer between the spatially separated nodes. These elements are laid out in a network (see Fig. 1a), with sufficient connectivity between modules to enable entanglement distribution and arbitrary qubit states transmission from one node to the other. At a circuit level, we analyze the generation and absorption efficiency at the nodes to be approximately 98%, achieving the state transfer process fidelity of 88% and entanglement between two nodes with a similar high fidelity of 91%. Crucially we show how well each component of the module can operate to meet the fault-tolerance threshold for the purification-based protocols^1,2^. These results indicate that this spin-photon module is consistent with the present experimental technology and can be directly used for scalable quantum computation with network architecture.

Recently, spin-photon strong coupling has been implemented in various quantum dot platforms, including single-electron spin qubit^15,16^, and three-electron spin qubit^17^. Compared to other kinds of spin qubits, the two-electron spin qubit studied here has several advantages: very long coherence times of up to 200^25^, universal single-qubit operation and two-qubit gate with high fidelity^23,26^, and rapid single-shot readout with high visibility^27^ have been demonstrated experimentally for such qubits. Remote entanglement has also been proposed in a spin-photon network^24^ and initial evidence for non-local coupling between two spins has been observed^18^. But a complete scheme of state transfer and remote entanglement is largely lacking. Here we develop a systematic framework for tackling the crucial challenges individually while constructing a cohesive design toward the spin-photon network architecture.

## Results

### Spin-photon module

We consider a module consisting of a spin-photon system as depicted in Fig. 1a. There are two elements in the module: the spin qubit in a semiconductor double quantum dot with two electrons, and single microwave photons in a superconducting resonator. By adjusting the potential difference of the two dots, the charge configuration (*n*_*L*_, *n*_*R*_) can be changed, where the notation (*n*_*L*_, *n*_*R*_) denotes the number of electrons in the left and right dot, respectively. The spin single-triplet qubit is defined as $\left|(1,1)S\right\rangle =\frac{1}{\sqrt{2}}(\left|\uparrow \downarrow \right\rangle -\left|\downarrow \uparrow \right\rangle )$ and $\left|(1,1){T}_{0}\right\rangle =\frac{1}{\sqrt{2}}(\left|\uparrow \downarrow \right\rangle +\left|\downarrow \uparrow \right\rangle )$, where ↑ and ↓ labels the spin up and down. Furthermore, we consider an auxiliary singlet state $\left|(0,2)S\right\rangle$ where two electrons are in the right dot, coupling the state $\left|(1,1)S\right\rangle$ via the tunneling *t*_*c*_^28–30^. As shown in Fig. 1b, we exploit the quantum dot in the charge degeneracy regime *ϵ* < < 0, in which the spin qubit has a Hamiltonian as:

1 $${H}_{q}=\frac{J}{2}{\tau }_{z}+\frac{\Delta B}{2}{\tau }_{x},$$

and offers maximum long spin coherence time and will be crucial for our scheme below. Here *τ*_*i*_ are the Pauli matrices defined in the basis of {$\left|(1,1){T}_{0}\right\rangle ,\left|(1,1)S\right\rangle$}, *ϵ* and *t*_*c*_ are the energy difference and tunneling energy between the two dots, *ΔB* is the magnetic field gradient, and the spin exchange energy $J=\frac{\epsilon }{2}+\sqrt{\frac{{\epsilon }^{2}}{4}+{t}_{c}^{2}}$ (see Fig. 1b and Supplementary Information).

A superconducting transmission line resonator of length *L* has the capacitance *C*_*r*_ and impedance *Z*_*r*_ per unit length. Thus the voltage of the resonator could be quantized as ${\widehat{V}}_{r}=k\sqrt{\frac{\hslash {\omega }_{k}}{L{C}_{r}}}({{ {\hat{a}} }}_{k}+{{ {\hat{a}} }}_{k}^{\dagger })$^31^, where ${\hat{a}}$ and $\widehat{{a}^{\dagger }}$ are the annihilation and creation operators, respectively. Usually, we omit the higher energy modes of the resonator and focus on the fundamental mode which associates with the energy splitting of the spin qubit. Thus the Hamiltonian of the resonator can be expressed as ${H}_{r}=\hslash {\omega }_{r}\left({{ {\hat{a}} }}^{\dagger }{ {\hat{a}} }+\frac{1}{2}\right)$ with the resonator frequency ${\omega }_{r}=\frac{k}{{C}_{r}{Z}_{r}}$ and the corresponding wave vector $k=\frac{\pi }{L}$. As shown in Fig. 1a, the resonator is coupled to the tunnel barrier of the two dots. The voltage of the resonator shifts the tunnel potential, thus the spin exchange energy is changed accordingly^24^. The coupling between the dot and the resonator is then given as:

2 $${H}_{c}={J}_{r}({ {\hat{a}} }+{{ {\hat{a}} }}^{\dagger }){\tau }_{z},$$

where *J*_*r*_ is the resonator-induced change of the spin exchange term (see Supplementary Information).

Putting things together, we can write the combined system as the following Hamiltonian in the basis of the eigenstates {$\left|e\right\rangle ,\left|g\right\rangle$}:

3 $$\begin{array}{lll}{H}_{t} & = & \frac{1}{2}\hslash {\omega }_{q}{\widehat{\sigma }}_{z}+\hslash {\omega }_{r}\left({{ {\hat{a}} }}^{\dagger }{ {\hat{a}} }+\frac{1}{2}\right)-\hslash g\ \sin 2\theta ({ {\hat{a}} }+{{ {\hat{a}} }}^{\dagger }){\widehat{\sigma }}_{x}\\  &  & +\,\hslash g\cos 2\theta ({ {\hat{a}} }+{{ {\hat{a}} }}^{\dagger }){\widehat{\sigma }}_{z}.\end{array}$$

Here ${\widehat{\sigma }}_{i}$ are the Pauli matrices defined in the basis of {$\left|e\right\rangle ,\left|g\right\rangle$}: $\left|g\right\rangle =\cos \theta \left|(1,1){T}_{0}\right\rangle +\sin \theta \left|(1,1)S\right\rangle$ and $\left|e\right\rangle =-\sin \theta \left|(1,1){T}_{0}\right\rangle +\cos \theta \left|(1,1)S\right\rangle$, the spin level splitting is ${\omega }_{q}=\sqrt{{J}^{2}+\Delta {B}^{2}}$, the mixing angle is $\theta =\frac{1}{2}\arctan \left(\frac{\Delta B}{J}\right)$, and the coupling strength is $g=\frac{{J}_{r}}{2\hslash }$ (see Supplementary Information). When the coupled system is in the large detuning regime *Δ* = *ω*_*q*_ − *ω*_*r*_ > > *g*, the energy level spectrum is shown in Fig. 1c.

Then we consider the system in the dressed states picture with the eigenstates ${\left|\pm \right\rangle }_{n}$ and eigenenergies ${E}_{n}^{\pm }$ (see Supplementary Information for detailed formulas). Now we analyze how to obtain a tunable qubit-resonator coupling by applying an external microwave pulse. The Hamiltonian for the driven system is given as: $H={H}_{t}+\hslash [V(t){\widehat{\sigma }}_{+}{e}^{-i{\omega }_{d}t}+{V}^{* }(t){\widehat{\sigma }}_{-}{e}^{i{\omega }_{d}t}]$. Here the microwave pulse with the driving frequency *ω*_*d*_ contains two slowly varying quantities: *V*(*t*) = *V*_0_(*t*)*e*^*iϕ*(*t*)^, the amplitude *V*_0_(*t*) and the phase *ϕ*(*t*), which play a central role in generating shaped single photons. Then we assume the frequency of the driving field is about $\frac{({E}_{2}^{+}-{E}_{0})}{2}\approx \hslash \left({\omega }_{r}+\frac{\Delta }{2}\right)$, thus the ground state $\left|g0\right\rangle$ and the excited state ${\left|+\right\rangle }_{2}$ can be resonance with two-photon process, as illustrated in Fig. 1c. In the subspace of five relevant states {$| g0\rangle ,{| -\rangle }_{1},{| +\rangle }_{1},{| +\rangle }_{2},{| +\rangle }_{3}$}, the interaction Hamiltonian can be described as:

4 $$\begin{array}{lll}{H}_{int} & = & \sum _{s=\pm }\sum _{{n}_{i},{n}_{f}=0,...3}\hslash {V}_{if}{\left|{s}_{f}\right\rangle }_{{n}_{f}{n}_{i}}\left\langle {s}_{i}\right|{e}^{i({E}_{{n}_{i}}^{s}-{E}_{{n}_{f}}^{s}-\hslash {\omega }_{d})t/\hslash }\\  &  & +\,H.c.\end{array}$$

where *V*_*if*_ are the transitions constants between the relevant states, ${\left|s\right\rangle }_{n}$ and ${E}_{n}^{s}$ are the eigenstates and eigenenergies, respectively (see Supplementary Information).

Due to the system in the large detuning, the state ${\left|+\right\rangle }_{2}$ is very close to $\left|e1\right\rangle$, thus the temporal evolution of the system can be calculated in the basis {$\left|g0\right\rangle ,\left|e1\right\rangle$}. When working in the weak driving field, removing the rapidly oscillating terms and using the time-averaging method^32^, the effective Hamiltonian of the spin-photon module is redefined as:

5 $${H}_{mod}=\hslash ({g}_{eff}(t)\left|g0\right\rangle \left\langle e1\right|+H.c.)+H{\prime} $$

where the transition between the states $\left|g0\right\rangle$ and $\left|e1\right\rangle$ has an effective coupling strength *g*_*eff*_(*t*), and the remaining term $H{\prime}$ is the ac Stark shift. In the dispersive regime, we can easily obtain ${g}_{eff}(t)\approx \frac{4gV{(t)}^{2}}{{\Delta }^{2}}$ using the perturbation theory. From the expression of *g*_*eff*_(*t*), we find the effective coupling strength *g*_*eff*_(*t*) ≪ *g* since the driving strength *V*(*t*) is much smaller than the detuning *Δ*. As a distinctive feature of this scheme, we utilize long spin coherence time, which ensures the shaped single photons can be generated ($\sim \frac{\pi }{{g}_{eff}}$) within the qubit coherence time.

### Generation of shaped single photons

In the previous schemes^33,34^, one generates the single photons by changing the qubit-resonator coupling strength. However, it can not be realized in the quantum dot system directly without additional tuning circuits. In contrast, here we explore a spin-photon module to control the transmission process from the state $\left|g0\right\rangle$ to the state $\left|e1\right\rangle$. When the system is in the state $\left|e1\right\rangle$, it decays into the state $\left|e0\right\rangle$ due to the photon emission. As illustrated in Fig. 1d, the system is trapped in the state $\left|e0\right\rangle$ since the state $\left|e0\right\rangle$ is off resonant to the state $\left|g1\right\rangle$, ensuring the emitted photon a single photon state. Thus, by adjusting the amplitude and the phase of the driving microwave pulse in time, we can manipulate the temporal shape of the emitted single photons. Furthermore, more advanced control method can be easily integrated with this microwave pulse, which opens a toolbox for spin-photon network architecture.

**Figure 2:**
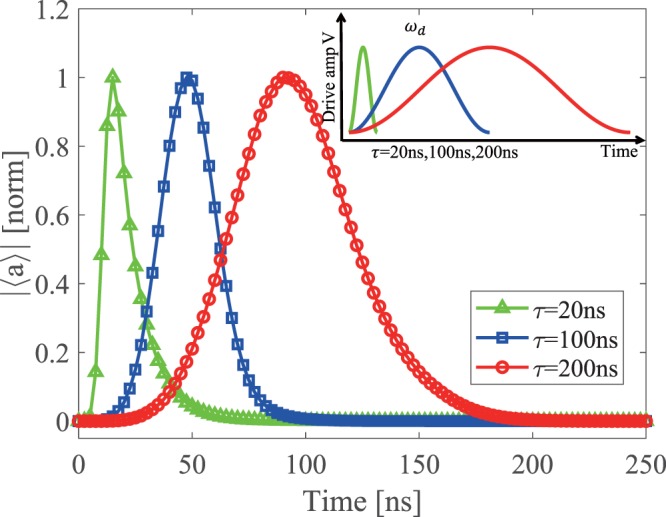
Microwave controlled generation of shaped single photons. The normalized amplitude of the obtained photon fields as a function of time (see text for details). Three driving pulses with durations *τ* and amplitudes *V*_0_ are shown in the inset.

As shown in Fig. 2, we select the driving pulse as:

6 $$V(t)=\hslash {V}_{0}{\sin }^{2}(\pi t/\tau ){e}^{i\phi (t)}$$

where *V*_0_ is the maximum amplitude, *τ* is the duration time, and *ϕ*(*t*) is the phase. The phase *ϕ*(*t*) changes with time to compensate for the ac Stark shift caused by the amplitude, making the phase of the emission photon field constant during the pulse. Compared with other driving pulses such as Gaussian function, this pulse has two advantages: simple form and no need for cutoff.

In order to demonstrate the shape of the photon field is fully controllable, the spin qubit is prepared as $(\left|g\right\rangle +\left|e\right\rangle )/\sqrt{2}$ corresponding to the photon state $(\left|0\right\rangle +\left|1\right\rangle )/\sqrt{2}$. Contrast to the Fock state $\left|1\right\rangle$, the superposition state $(\left|0\right\rangle +\left|1\right\rangle )/\sqrt{2}$ has a nonzero photon field $\left\langle a\right\rangle$ which characterizes the shape of the single photon. We perform the numerical simulations of the spin-photon module including the photon decay and the spin qubit decoherence. The master equation is described as:

7 $$\dot{\rho }=-\frac{i}{\hslash }[{H}_{mod},\rho ]+\kappa D[a]\rho +{\gamma }_{1}D[{\sigma }_{-}]\rho +\frac{{\gamma }_{\phi }}{2}D[{\sigma }_{z}]\rho ,$$

where $D[L]\rho =L\rho {L}^{\dagger }-\frac{1}{2}({L}^{\dagger }L\rho +\rho {L}^{\dagger }L)$, *ρ* is the density matrix of the system, *κ* is the photon decay rate of the resonator, *γ*_1_ and *γ*_*ϕ*_ is the relaxation rate and dephasing rate of the spin single-triplet qubit, respectively.

We are interested in the effects of duration *τ* and maximum amplitude *V*_0_ on the photon waveform. By changing the key parameters *τ* and *V*_0_, we can maximize the symmetry of the photon shape (see Supplementary Information for detailed investigations). Figure 2 show the photon field $\left\langle a\right\rangle$ in three situations of *τ* and *V*_0_ with high symmetry: the durations of the pulses are *τ* = 20, 100, 200 ns and the corresponding amplitudes are *V*_0_/2*π* = 0.9, 0.98, 0.73 GHz, respectively. Other parameters we used are experimental reported numbers: *ω*_*r*_ = 2*π* × 6.2 GHz^15–17^, *κ* = 2*π* × 30 MHz, *g* = 2*π* × 30 MHz (see Supplementary Information for the estimation of this quantity), *ω*_*q*_ = 2*π* × 9.0 GHz^28^, *T*_1_ = 1/*γ*_1_ = 10 *μ*s, and *T*_*φ*_ = 1/*γ*_*φ*_ = 1 *μ*s. We note that *T*_*φ*_ is in the order of *μ*s for singlet-triplet qubits in Si^35–37^ and GaAs^23,38,39^ quantum dots. We also consider the possibility of applying dynamical decoupling pulses to our scheme, as they dramatically improve coherence time (see Supplementary Information for detailed discussions).

For a shorter pulse (*τ* = 20ns), the symmetry of the photon field is much worse than the longer pulse because the driving pulse is too short to transfer the state from $\left|g0\right\rangle$ to $\left|e1\right\rangle$ completely. Therefore, the high symmetry can be obtained only when the generation time of the photon field is much longer than the resonator rise time *T*_*r*_ = 1/*κ* ≈ 6 ns. In addition, the phase of the photon is also symmetric in time since the driving pulse phase *ϕ*(*t*) keeps the phase of the photon field constant during the operation, which enables the re-absorption of the single photon as outlined below. Overall, we have demonstrated that the single photons with controllable shape can be prepared successfully using the spin-photon module. This scheme can also prepare a multipeaks photon and the phase of its peaks is tunable, which can be used to encode quantum information over time bins (see Supplementary Information for further discussions).

### Deterministic state transfer between remote nodes

To explore the universal functions of the spin-photon module, here we connect two nodes A and B with a coaxial line as shown in Fig. 1a, allowing the photonic links within this configuration. Applying a pulse to the spin qubit in node A, a single photon emits with time symmetric shape. Then the itinerant photon absorbs in node B using a pulse with time reversed amplitude and phase, which involves the state reverse process. The Hamilontian for a simple network is given as^40^:

8 $$\begin{array}{lll}{H}_{net} & = & \sum _{i=A,B}(\hslash {g}_{eff}^{i}(t){\left|g0\right\rangle }_{ii}\left\langle e1\right|+H.c.+{H{\prime} }_{i})\\  &  & +\,i\hslash \frac{\sqrt{{\kappa }^{A}{\kappa }^{B}{\eta }_{t}}}{2}({{ {\hat{a}} }}_{A}{{ {\hat{a}} }}_{B}^{\dagger }-{{ {\hat{a}} }}_{A}^{\dagger }{{ {\hat{a}} }}_{B})\end{array}$$

where the subscript *i* = *A*, *B* denotes node A and node B, *κ*^*A*^(*κ*^*B*^) represents the photon emission rate from node A(B) and *η*_*t*_ is the photon transmission efficiency between two nodes. We put the Hamiltonian into the master equation:

9 $$\begin{array}{lll}\dot{\rho } & = & -\frac{i}{\hslash }[{H}_{net},\rho ]\\  &  & +\,\sqrt{{\eta }_{t}}D[\sqrt{{\kappa }^{A}}{{ {\hat{a}} }}_{A}+\sqrt{{\kappa }^{B}}{{ {\hat{a}} }}_{B}]\rho \\  &  & +\,\sqrt{(1-{\eta }_{t})}D[\sqrt{{\kappa }^{A}}{{ {\hat{a}} }}_{A}+\sqrt{{\kappa }^{B}}{{ {\hat{a}} }}_{B}]\rho \\  &  & +\,\sum _{i=A,B}\{{\kappa }_{int}^{i}D[{{ {\hat{a}} }}_{i}]\rho +{\gamma }_{l}^{i}D[{\sigma }_{-}^{i}]\rho +{\gamma }_{\varphi }^{i}D[{\sigma }_{z}^{i}]\rho \}\end{array}$$

where the parameters are followed the definitions in Eq. (7) (see Supplementary Information).

**Figure 3:**
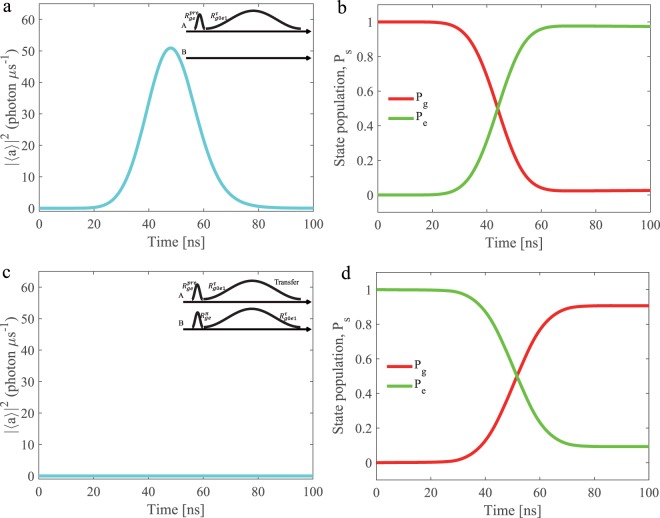
Universal quantum network modules. (**a**) The emitted photon field amplitude, with node A initially in $(\left|g0\right\rangle +\left|e0\right\rangle )$/$\sqrt{2}$. (**b**) The populations of the spin states during the photon emitting process, with node A initially in $\left|g0\right\rangle$. (**c**) The residual photon field in node B during the photon absorption process, with node A and B prepared in $(\left|g0\right\rangle +\left|e0\right\rangle )$/$\sqrt{2}$ and $\left|e0\right\rangle$. (**d**) The populations of the spin states in node B during the receiving process, with node A and B prepared in $\left|g0\right\rangle$ and $\left|e0\right\rangle$. The inserts in these figures show the applying pulses.

Firstly, we consider the single photon emitting process of node A as shown in Fig. 3a. We initialize the spin qubit on node A to the state $(\left|g0\right\rangle +\left|e0\right\rangle )$/$\sqrt{2}$. Then we generate a single photon with symmetric shape using a modulated microwave pulse *V*(*t*), which induces an excitation transfer process ${R}_{g0e1}^{\tau }$ as $(\left|e1\right\rangle +\left|e0\right\rangle )/\sqrt{2}$. We also change the phase of the pulse *V*(*t*) to compensate the ac Stark shift caused by the amplitude of the pulse. Fig. 3a shows the emitted photon field of node A over the time. Similarly, we prepare the initial spin state on node A as $\left|g0\right\rangle$ and implement the driving process as ${R}_{g0e1}^{\tau }$. Fig. 3b shows the populations of the spin states $\left|g0\right\rangle$ and $\left|e1\right\rangle$ of node A during the process. We find that the state $\left|g0\right\rangle$ evolves to the state $\left|e1\right\rangle$ smoothly and the population of the state $\left|e1\right\rangle$ ultimate reaches *P*_*e*_ = 97.4%. From these simulation results we extract the emission efficiency as high as 98%.

Secondly, we study the spin state transfer from node A to node B. Fig. 3c shows the pulse scheme used for emitting a single photon from node A and re-absorbing it at node B. We prepare the spin qubit state of the node A and node B as $(\left|g0\right\rangle +\left|e0\right\rangle )$/$\sqrt{2}$ and $\left|e0\right\rangle$, respectively. A driving pulse of node A creates the state $(\left|e1\right\rangle +\left|e0\right\rangle )$/$\sqrt{2}$. Then a reverse pulse on node B generates the state $(\left|g0\right\rangle +\left|e0\right\rangle )$/$\sqrt{2}$, with the resonator returning to its ground state $\left|0\right\rangle$. We show the photon field during the absorption process of node B in Fig. 3c. Here we use the photon transmission efficiency from node A to node B as *η*_*t*_ = 92%, because there exists an extra photon loss when a single photon travels through the coaxial line. Similarly, we prepare the initial state of spin qubit in node A and node B as $\left|g0\right\rangle$ and $\left|e0\right\rangle$, respectively. We employ the same state transfer process and show the population of spin state of node B in Fig. 3d. We can observe that the population of state $\left|g0\right\rangle$ rises smoothly and stabilises at *P*_*e*_ = 90.7%, which represents the efficiency of the state transfer using this scheme.

**Figure 4:**
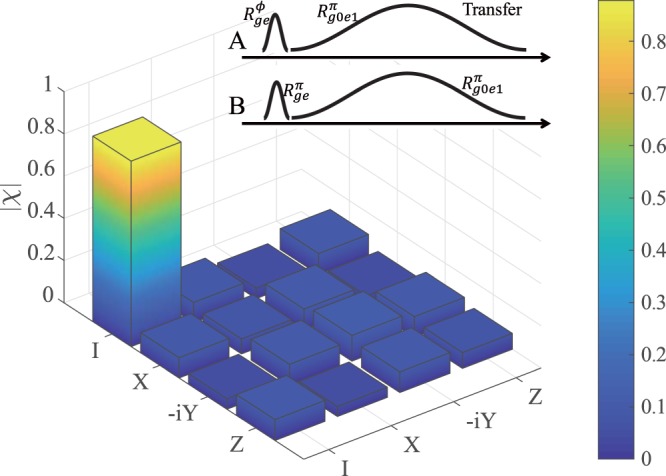
Quantum state transfer between two network nodes. Inset: The state transfer between node A and node B can be achieved via exchange of a single photon, with the quantum information encoded in the spin states and the photon states. Main: The process matrix of the state transfer in the basis {*I*, *X*, − *iY*, *Z*} using the quantum process tomography, and the fidelity reaches *F*_*p*_ = 87.8%.

To completely evaluate our scheme used to transfer a spin qubit state between remote nodes, we calculate the process fidelity by the quantum process tomography^41,42^. We prepare four mutually unbiased spin qubit states of node A as $\left|g0\right\rangle$, $\left|e0\right\rangle$, $(\left|g0\right\rangle +\left|e0\right\rangle )/\sqrt{2}$ and $(\left|g0\right\rangle +i\left|e0\right\rangle )/\sqrt{2}$, and the state of node B as $\left|e0\right\rangle$ simultaneously. Then we transfer the above states to node B and reconstruct the transfer process matrix *χ*, as shown in Fig. 4. The fidelity of the state transfer process can be determined as *F*_*p*_ = *tr*(*χχ*_*ideal*_) = 87.8%, which is higher than the classical threshold^7^ and can be used for state entanglement further. Ultimately, we demonstrate that the spin-photon modules form universal function nodes, which is the building blocks for a quantum network architecture.

### Preparation of remote entanglement

To show the power of the present spin-photon module, we explore the deterministic preparation of remote entanglement states between two spin qubits. Different from the previous solutions for generating entanglement states, our scheme consists three steps: Step 1, the spin qubits on node A and node B are prepared in the states $\left|g\right\rangle$ and $\left|e\right\rangle$, respectively. Step 2, a driving pulse ${R}_{g0e1}^{\pi /2}$ is applied on the spin qubit in node A which emits a single photon to node B. Step 3, we apply a time reversing pulse ${R}_{g0e1}^{\pi /2}$ on the spin qubit in node B which absorbs the single photon travelling from node A. Therefore the remote Bell type entanglement state $\left|{\Psi }^{+}\right\rangle =({\left|g,e\right\rangle }_{AB}+{\left|e,g\right\rangle }_{AB})/\sqrt{2}$ is successful prepared.

**Figure 5:**
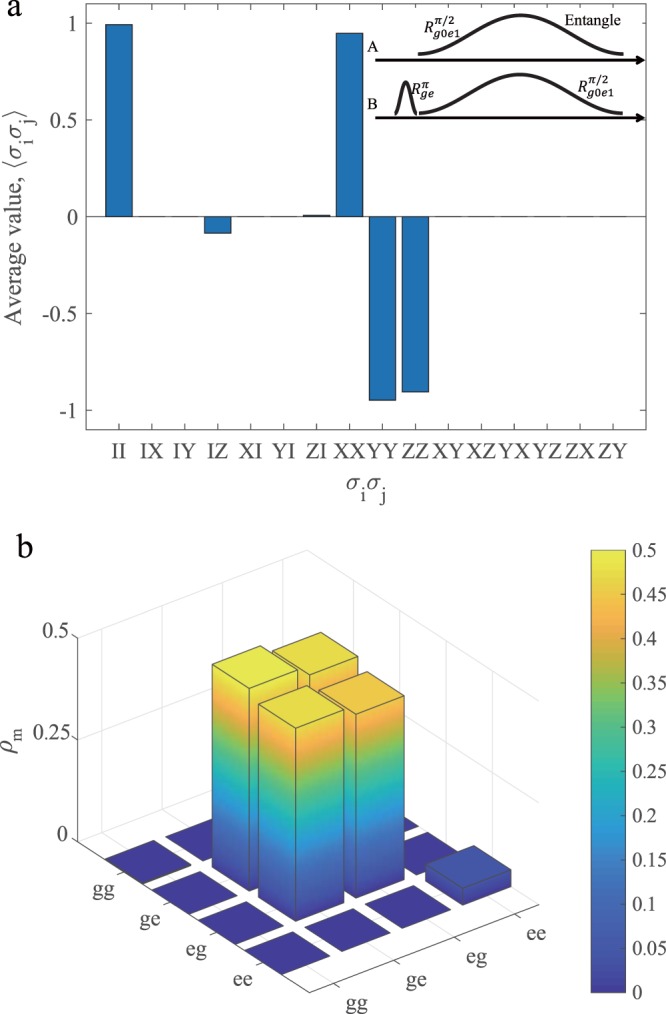
Remote entanglement of two nodes. (**a**) The average value of the Pauli operators $\left\langle {\sigma }_{i}{\sigma }_{j}\right\rangle$ for the spin qubits in two nodes. The pulses are shown in the inset. (**b**) The density matrix *ρ*_*m*_ of the generated remote entangled state and the state fidelity is *F*_*s*_ = 90.8% relative to the ideal Bell state.

Considering the photon loss and the spin qubit decoherence, we numerically simulate the dynamics of the whole system using the master equation. Then the calculated two-qubit density matrix *ρ*_*m*_ is expressed using the quantum state tomography. Fig. 5a shows the average value of the Pauli operators $\left\langle {\sigma }_{i}{\sigma }_{j}\right\rangle$ of the spin qubits in two nodes. Fig. 5b is the reconstructed density matrix *ρ*_*m*_ of the remote entanglement state using our scheme. It is not difficult to find that the state fidelity is ${F}_{s}=\left\langle {\Psi }^{+}\right|{\rho }_{m}\left|{\Psi }^{+}\right\rangle =90.8 \%$ contrast to the ideal Bell state. Overall, our scheme is able to the generation of deterministic remote entanglement with a high fidelity, which is potential to be used for various quantum information protocols in a network architecture.

### Benchmarking the spin-photon network

As shown in Fig. 1a, the proposed module supports a network architecture in a fault-tolerant manner. For a network architecture, we consider the following error model: (1) preparation, operation and measurement process within a module, (2) Bell pair creation process between different modules. We take the error probability to be *p*_*node*_ and *p*_*link*_ for these two sources of error, respectively. The highly flexible nature of the presented module makes it amenable to the use of a variety of topological error correction protocols. Having specified the entanglement-purification protocols^1,2^, the error threshold has been established numerically and the results are displayed in Table 1. Given a simple module design, the threshold of ${p}_{link}^{th}\approx 10 \%$ for inter-module links is found. Our proposed error rate for remote entanglement is  ≈ 9.2%, which is sufficiently below the threshold to allow suppression of errors. Such implementation presents a milestone on the path toward fault-tolerant quantum information processing. Moreover, by increasing the coherence time of the spin qubit and decreasing the photon loss between the two nodes, we could enhance the fidelity better to significantly reduce the overhead (see Supplementary Information for more discussions).

**Table 1:** The threshold of tolerable error rates for intra- and inter-module operations in a network architecture. Our scheme is already or approaching the desired regime.

	${p}_{node}^{th}$	${p}_{link}^{th}$
NN protocol^1^	0.86%	10%
our architecture	1%	9.2%

## Conclusions

In summary, we propose a spin-photon module as building blocks of a scalable network architecture for quantum information processing. The controllable coupling between spin qubits and microwave photons allows for long distance state transfer mediated by photons. This coupling can then be employed to perform entangling two-qubit states between spins separated in remote nodes. Combination of long coherence time on a semiconductor chip and full tunability of the spin-photon interface is one characteristic feature of the spin-photon module, which makes it a strong candidate for scalable quantum network platform. Given the threshold of purification-based quantum error correction protocols, the error rate of our scheme is sufficiently below the threshold to allow significant suppression of errors with present technology. Moreover, the possibility of creating a network of spin qubits with engineered coupling and designed geometries is very useful for quantum simulation of interacting quantum many-body systems.

## Methods

### Effective Hamiltonian of the spin-photon module

For the spin-qubit part, we use the adiabatic elimination method to yield the singlet-triplet Hamiltonian Eq. (1). For the coupling between the spin and the resonator, we use the Heitler-London method to model the tunnel barrier of the double quantum-dot and its change by the voltage of the resonator. Then the spin-photon hybrid system can be conveniently analyzed in the dressed states picture. When a microwave pulse is applied, the hybrid system can be reduced in the subspace of only five dressed states as Eq. (4). Using the time-averaging method to remove the rapidly oscillating terms, the evolution of the spin-photon module can be described as an effective Hamiltonian between the states $\left|g0\right\rangle$ and $\left|e1\right\rangle$ given by Eq. (5). The complete expressions and the detailed derivations of the effective Hamiltonian can be found in the Supplement Information.

### Simulations of master equations

Simulations of the spin-photon network are carried out using the master equation approach. We obtain the corresponding evolution for each of the schemes (sending, receiving the single photons, and remote entanglement between two nodes) by numerically solving the appropriate time-dependent master equations (Eqs. (7) and (9)). The numerical simulations included the effects of decoherence expected for spin qubits, as well as errors in the photon channel, with physical parameters as indicated. More details and discussions can be found in the Supplement Information.

## Supplementary information

Supplementary Information.
