# Supplementary material for: Spin-photon module for scalable network architecture in quantum dots
Source: Sci Rep. 2020 Mar 19;10:5063. doi: 10.1038/s41598-020-61976-2 (PMC7081348; doi:10.1038/s41598-020-61976-2)
Supplement: Supplementary file 1 — Supplementary Information. [file 41598_2020_61976_MOESM1_ESM.pdf]

Supplementary Information for
Spin-photon module for scalable network architecture in quantum dots

Xing-Yu Zhu, Tao Tu, Ao-Lin Guo, Zong-Quan Zhou, Guang-Can Guo, and Chuan-Feng Li

Section S1: Spin singlet-triplet qubit

We consider a CQED-like system combined a spin qubit in semiconductor double-quantum dot and a superconducting transmission line resonator. We use the notation (n_L, n_R) to denote the number of electrons in the left and right dot, and S and T to label the spin singlet and triplet states. The Hamiltonian of the spin qubit in the basis $\{|(1,1)T_0\rangle, |(1,1)S\rangle, |(0,2)S\rangle\}$ is described as:

$$H_{QD} = \begin{pmatrix} 0 & \Delta B/2 & 0 \\ \Delta B/2 & 0 & t_c \\ 0 & t_c & -\epsilon \end{pmatrix}, \tag{S1}$$

and the energy level diagram is shown in Fig. 1b of the main text. Here $\Delta B = g\mu_B dB$, dB is the magnetic field gradient between the two quantum dots [1–3], ϵ and t_c are the energy difference and tunneling energy between the two dots, respectively.

We may adiabatic eliminate the doubly occupied state $|(0,2)S\rangle$ when the detuning ϵ between the two dots is much larger than the operation frequency scale. To do so, we start by writing the Hamiltonian for the states $|(1,1)S\rangle$ and $|(0,2)S\rangle$:

$$H_S = \begin{pmatrix} 0 & t_c \\ t_c & -\epsilon \end{pmatrix}. \tag{S2}$$

Diagonalizating the above matrix, we obtain two adiabatic eigenstates:

$$\begin{aligned} |D\rangle &= \cos\alpha\,|(1,1)S\rangle + \sin\alpha\,|(0,2)S\rangle\,, \\ |U\rangle &= -\sin\alpha\,|(1,1)S\rangle + \cos\alpha\,|(0,2)S\rangle\,. \end{aligned} \tag{S3}$$

Here the adiabatic angle is $\alpha = \arctan(\frac{2t_c}{-\epsilon+\sqrt{\epsilon^2+4t_c^2}})$, and the adiabatic eigenenergies are:

$$\begin{aligned} E_D &= t_c \tan(\alpha)/2, \\ E_U &= t_c \tan(\alpha - \frac{\pi}{2})/2, \end{aligned} \tag{S4}$$

respectively. Following the adiabatic elimination method [4], the eigenstate $|D\rangle$ becomes $|(1,1)S\rangle$ in the charge degeneracy region $\epsilon << 0$, while the eigenstate $|D\rangle$ develops to $|(0,2)S\rangle$ in the charge biased region $\epsilon >> 0$. Thus, the spin qubit has an effective Hamiltonian in the basis of $\{|(1,1)T_0\rangle, |(1,1)S\rangle\}$:

$$H_q = \begin{pmatrix} 0 & \Delta B/2 \\ \Delta B/2 & -J \end{pmatrix}, \tag{S5}$$

with the spin exchange energy $J = E_D$ depending on ϵ and t_c:

$$J = \frac{\epsilon}{2} + \sqrt{\frac{\epsilon}{4}^2 + t_c^2}. \tag{S6}$$

Section S2: Coupling between the spin qubit and the resonator

On the other hand, following the Heitler-London method [5], the double-quantum dot can be modeled as two dots separated by a parabolic tunnel barrier

$$V_t = V_0 - \frac{m\omega_0^2 x^2}{4}, \tag{S7}$$

where the height is $V_0 = m\omega_0^2 a_0^2/8$, x is the position of the potential, a_0 is the position of the center of the quantum dot. As illustrated in Fig. 1a of the main text, a superconducting transmission line resonator is coupled to the interdot tunnel barrier of the double-quantum dot. The potential of the tunnel barrier is changed by the voltage of the resonator V_r as:

$$\Delta V_t = eV_r \frac{x^2}{a_0^2}. \tag{S8}$$

As a result, the spin exchange energy is changed accordingly, and the interaction between the spin qubit and the resonator is given as:

$$H_c = J_r(\hat{a} + \hat{a}^\dagger)\tau_z \tag{S9}$$

where τ_z is the Pauli matrix in the basis of $\{|(1,1)T_0\rangle, |(1,1)S\rangle\}$, $\hat{a}^\dagger$ and $\hat{a}$ are the creation and annihilation operators of the resonator, respectively. The resonator-induced spin exchange term J_r can be calculated as [5]:

$$\begin{aligned} J_r =& \frac{1}{2}\sum_{i=1,2}[\langle(1,1)T_0|\,\Delta V_t(x_i)\,|(1,1)T_0\rangle \\ &- \langle(1,1)S|\,\Delta V_t(x_i)\,|(1,1)S\rangle] \\ =& eV_r \sinh[\frac{16V_0(\omega_0^2 + 2\omega_L^2)}{\hbar\omega_0^2\sqrt{\omega_0^2+\omega_L^2}}]^{-1}, \end{aligned} \tag{S10}$$

where $\omega_L = eB/2m$ is the Larmor frequency and B is the magnetic field.

Section S3: Effective Hamiltonian of the combined spin-photon system

Taking into account both the spin qubit and the resonator, we obtain the total Hamiltonian for the combined system:

$$\begin{aligned} H_t &= H_q + H_r + H_c \\ &= \frac{J}{2}\tau_z + \frac{\Delta B}{2}\tau_x + \hbar\omega_r(\hat{a}^\dagger\hat{a} + \frac{1}{2}) + J_r(\hat{a} + \hat{a}^\dagger)\tau_z. \end{aligned} \tag{S11}$$

Working in the eigenbasis of $|e\rangle$ and $|g\rangle$ of the spin qubit, the total Hamiltonian takes the form:

$$\begin{aligned} H_t =& \frac{1}{2}\hbar\omega_q\hat{\sigma}_z + \hbar\omega_r(\hat{a}^\dagger\hat{a} + \frac{1}{2}) - \hbar g \sin 2\theta(\hat{a} + \hat{a}^\dagger)\hat{\sigma}_x \\ &+ \hbar g \cos 2\theta(\hat{a} + \hat{a}^\dagger)\hat{\sigma}_z. \end{aligned} \tag{S12}$$

This is exactly the Eq. (3) of the main text. Here, the operators $\hat{\sigma}_{x,y,z}$ are the Pauli matrices in the eigenbasis of $|e\rangle$ and $|g\rangle$, ω_q and ω_r represent the frequency of the spin qubit and the resonator, respectively. The coupling strength is $g = \frac{J_r}{2\hbar}$ and the mixing angle is $\theta = \frac{1}{2}\arctan(\frac{\Delta B}{J})$. In particular, when we adjust the spin qubit to the charge degeneracy region $\epsilon << 0$, leading to $\theta \to \frac{\pi}{4}$, we can represent the Hamiltonian (S12) as:

$$H_t = \frac{1}{2}\hbar\omega_q\hat{\sigma}_z + \hbar\omega_r(\hat{a}^\dagger\hat{a} + \frac{1}{2}) - \hbar g(\hat{a} + \hat{a}^\dagger)\hat{\sigma}_x. \tag{S13}$$

We note that the above equation reduces to the Jaynes-Cummings Hamiltonian in the rotating wave approximation [9].

Using the above formula (S10) and (S12), we can estimate the realizable coupling strength between the spin qubit and the resonator. The parameters are chosen for experiments in GaAs quantum dots with confining potential $\hbar\omega_0 = 4.5$ meV, resonator induced voltage $V_r = 1$ μV [5–8]. At magnetic field $B = 1.5$ T, we have $J_r = 0.3$ μeV. Thus we obtain the coupling strength between the spin qubit and the photon as $g = 2\pi \times 30$ MHz which is used in the main text. Given that the strength of the coupling can reach several tens of MHz, and the spin qubit reaches the long decoherence time $T_2 = 1$ μs, the system reaches the strong coupling regime.

Section S4: The driven spin-photon module

We apply a driving microwave pulse on the coupled spin-photon system, and present it as:

$$H_d = \hbar(V(t)\hat{\sigma}_+ e^{-i\omega_d t} + V^*(t)\hat{\sigma}_- e^{i\omega_d t}) \tag{S14}$$

where $V(t) = V_0(t)e^{i\phi(t)}$ and ω_d are the complex strength and frequency of the microwave pulse, respectively. Taking into account all the terms gives a Hamiltonian:

$$H = H_t + H_d. \tag{S15}$$

Then we consider the system in the dressed states picture [9]. Diagonlizing the Hamiltonian H_t directly, we can obtain the eigenstates of the hybrid spin-photon system:

$$\begin{aligned}\left|+\right\rangle_n &= \sin\phi_n \left|g\right\rangle \left|n\right\rangle + \cos\phi_n \left|e\right\rangle \left|n-1\right\rangle, \\ \left|-\right\rangle_n &= \cos\phi_n \left|g\right\rangle \left|n\right\rangle - \sin\phi_n \left|e\right\rangle \left|n-1\right\rangle,\end{aligned} \tag{S16}$$

with the corresponding eigenenergies:

$$E_n^{\pm} = \hbar(n\omega_r \pm \sqrt{(\Delta/2)^2 + ng^2}). \tag{S17}$$

Here $\Delta = \omega_q - \omega_r$ is the energy detuning between the spin qubit and the resonator, n denotes the resonator excitation, and the dressed angle is defined as $\phi_n = 2\arctan(\frac{2\sqrt{n}g}{\Delta})$. In addition, the eigenenergy of the ground state $|g0\rangle$ is $E_0 = -\hbar\Delta/2$.

In the eigenbasis of $\left|+\right\rangle_n$ and $\left|-\right\rangle_n$, the Hamiltonian of (S15) can be rewritten as:

$$\begin{aligned}H = &-\frac{\hbar\Delta}{2}\left|g0\right\rangle\left\langle g0\right| + \sum_{s=\pm}\sum_{n=1}^{\infty} E_n^s \left|s\right\rangle\left\langle s\right| \\ &+ \hbar(V(t)\widetilde{\sigma}_+ e^{-i\omega_d t} + V^*(t)\widetilde{\sigma}_- e^{i\omega_d t}),\end{aligned} \tag{S18}$$

with the operators $\widetilde{\sigma}_+ = \widetilde{\sigma}_-^{\dagger} = \sum_{n=0}^{\infty} \left|gn\right\rangle\left\langle en\right|$. In the rotating frame of the driving pulse with frequency ω_d, the energy level diagram is illustrated in Fig. 1c of the main text.

When the microwave frequency ω_d is chosen to be about $\frac{(E_2^+ - E_0)}{2} \approx \hbar(\omega_r + \frac{\Delta}{2})$, the system is driven by two-photon resonance between the two states $|g0\rangle$ and $\left|+\right\rangle_2$. The three dressed states $\left|\pm\right\rangle_1$ and $\left|+\right\rangle_3$ are detuned by $\Delta/2$ with respect to the driving frequency. We notice that as n increases, the state detuning also increases, which leads to the other dressed states, such as $\left|-\right\rangle_2$, $\left|-\right\rangle_3$ are detuned more than Δ. Therefore, we can consider the evolution of the system in the subspace of only five dressed states $\{\left|g0\right\rangle, \left|-\right\rangle_1, \left|+\right\rangle_1, \left|+\right\rangle_2, \left|+\right\rangle_3\}$. The interaction Hamiltonian can be described as:

$$\begin{aligned}H_{int} =& \hbar(V(t)\cos\phi_1 \left|+\right\rangle_1\left\langle g0\right| e^{i(\frac{E_1^+}{\hbar} - \frac{E_0}{\hbar} - \omega_d)t} \\ &+ V(t)\sin\phi_1 \left|-\right\rangle_1\left\langle g0\right| e^{i(\frac{E_1^-}{\hbar} - \frac{E_0}{\hbar} - \omega_d)t} \\ &+ V(t)\sin\phi_1\cos\phi_2 \left|+\right\rangle_{21}\left\langle +\right| e^{i(\frac{E_2^+}{\hbar} - \frac{E_1^+}{\hbar} - \omega_d)t} \\ &- V(t)\cos\phi_1\cos\phi_2 \left|+\right\rangle_{21}\left\langle -\right| e^{i(\frac{E_2^+}{\hbar} - \frac{E_1^-}{\hbar} - \omega_d)t} \\ &+ V(t)\sin\phi_2\cos\phi_3 \left|+\right\rangle_{32}\left\langle +\right| e^{i(\frac{E_3^+}{\hbar} - \frac{E_2^+}{\hbar} - \omega_d)t}) \\ &+ H.c.\end{aligned} \tag{S19}$$

which is the complete expression of Eq. (4) in the main text.

In the dispersive regime of the hybrid spin-photon system, the eigenenergies of the dressed states approximate to $E_n^{\pm} \approx \hbar(\omega_r \pm (\Delta/2 + ng^2/\Delta))$, and we can define the frequency of the driving pulse as $\omega_d \approx \omega_r + (\Delta/2 + ng^2/\Delta)$. For the weak driving pulse, using the time-averaging method [10] to remove the rapidly oscillating terms, the Eq. (S19) is reduced to an effective Hamiltonian of the spin-photon module:

$$H_{mod} = \hbar(g_{eff}(t)\left|g0\right\rangle\left\langle e1\right| + H.c.) + H'. \tag{S20}$$

Here, we represent the interaction between the states $|g0\rangle$ and $|e1\rangle$, because the energy level of $|e1\rangle$ is very close to the dressed state $\left|+\right\rangle_2$. The term H' includes small ac Stark shift induced by the driving pulse. The effective coupling coefficient is given by:

$$g_{eff} = V^2(t)\sin\phi_1\cos\phi_1\cos\phi_2(\frac{2}{\Delta} + \frac{1}{\frac{\Delta}{2} + \frac{2g^2}{\Delta}}). \tag{S21}$$

Due to the large detuning between the spin qubit and the resonator, the dressed angle becomes $\cos\phi_n \simeq 1$ and $\sin\phi_n \simeq \sqrt{n}g/\Delta$. Thus, the effective coupling coefficient can be expressed as:

$$g_{eff} \simeq \frac{4gV^2(t)}{\Delta^2}, \tag{S22}$$

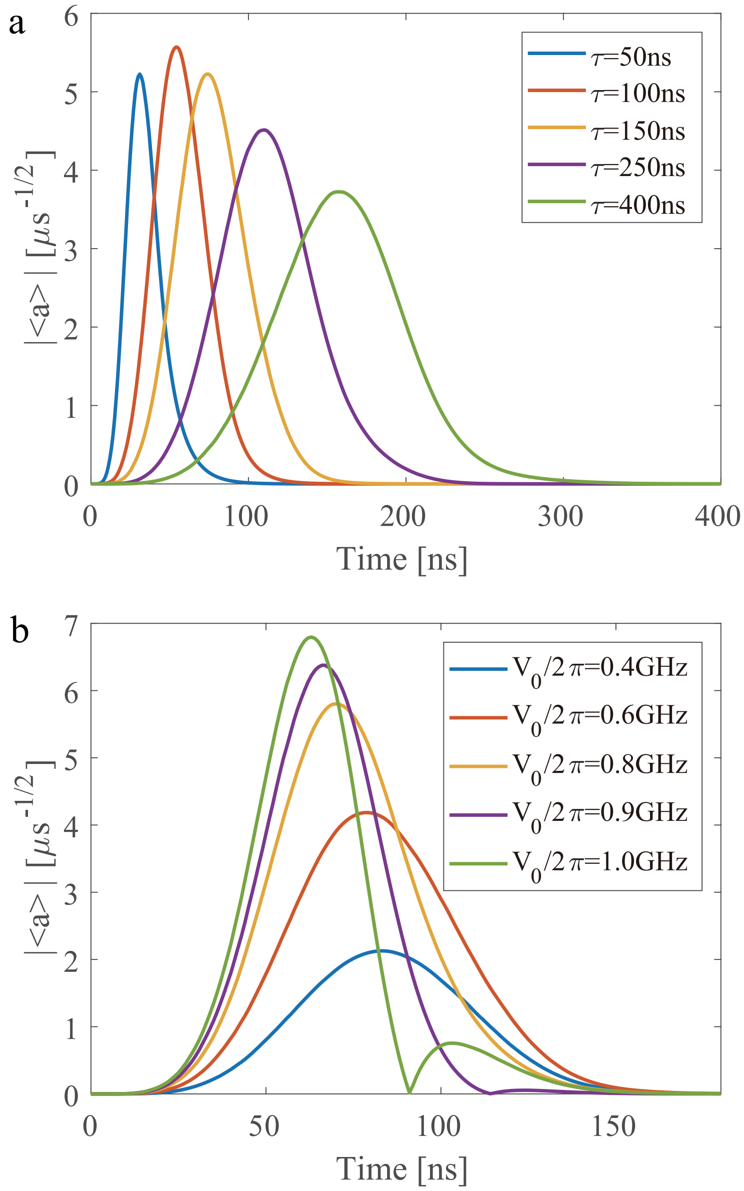

Fig. S1: The photon shape as a function of control parameters (a) The output photon field for various durations τ with fixed amplitude $V_0 = 2\pi \times 0.72$ GHz of the driving pulse. (b) The output photon field for different amplitudes V_0 with constant duration $\tau = 150$ ns of the driving pulse.

i.e., the result in the main text.

Section S5: The shape of the single photons

Here, we discuss the single photon shape controlled by a driving pulse with numerical simulations based on a master equation description of the spin-photon module:

$$\dot{\rho} = -\frac{i}{\hbar}[H_{mod}, \rho] + \kappa D[a]\rho + \gamma_1 D[\sigma_-]\rho + \frac{\gamma_\phi}{2} D[\sigma_z]\rho. \tag{S23}$$

Here $D[L]\rho = L\rho L^\dagger - \frac{1}{2}(L^\dagger L\rho + \rho L^\dagger L)$, ρ is the density matrix of the system, κ is the photon decay rate of the resonator, γ_1 and γ_ϕ is the relaxation rate and dephasing rate of the spin single-triplet qubit, respectively. We are interested in the effect of the amplitude V_0 and the duration τ of the driving pulse, and then explore the optimal parameters for the generation of the symmetric single photon state. Firstly, we prepare the spin qubit in the initial

state as $(|g\rangle + |e\rangle)/\sqrt{2}$ since the average photon field of the superposition state is nonzero in this case. Secondly, the driving pulses with a series of parameters V_0 and τ is applied to the spin-photon module. Finally, we calculate the output photon field to investigate the effect of V_0 and τ on the single photon shape.

To investigate the effect of the control parameters on the shape of the single photons, we fix the value of the amplitude $V_0 = 2\pi \times 0.72$ GHz and change the value of duration τ ranging from 50 ns to 400 ns. The results are shown in Fig. S1a. From the picture, we find that a larger duration τ leads to a longer photon field, while a shorter driving pulse, such as $\tau = 50$ ns, can not completely transfer the state from $|g0\rangle$ to $|e1\rangle$ and emission efficiency is worse.

We also consider the effect of the amplitude V_0 of the driving pulse, as shown in Fig. S1b. We keep the duration $\tau = 150$ ns and adjust the amplitude V_0 ranging from $2\pi \times 0.4$ GHz to $2\pi \times 1.0$ GHz. As the amplitude value increases, the shape of the single photon is narrowed. There arises a smaller peak near the single photon peak analogy to the Rabi oscillations, for the case of $V_0 = 2\pi \times 1.0$ GHz. In contrast, the weak driving pulse results a lower and fatter waveform of the photon field due to the incomplete state transfer between $|g0\rangle$ and $|e1\rangle$.

As a result, we can control the shape of the single photon state by adjusting the amplitude V_0 and duration τ of the driving pulse. Furthermore, we find the optimal values of V_0 and τ to generate the symmetric single photons, as shown in Fig. 2 of the main text.

Section S6: Generation of multipeaked photons

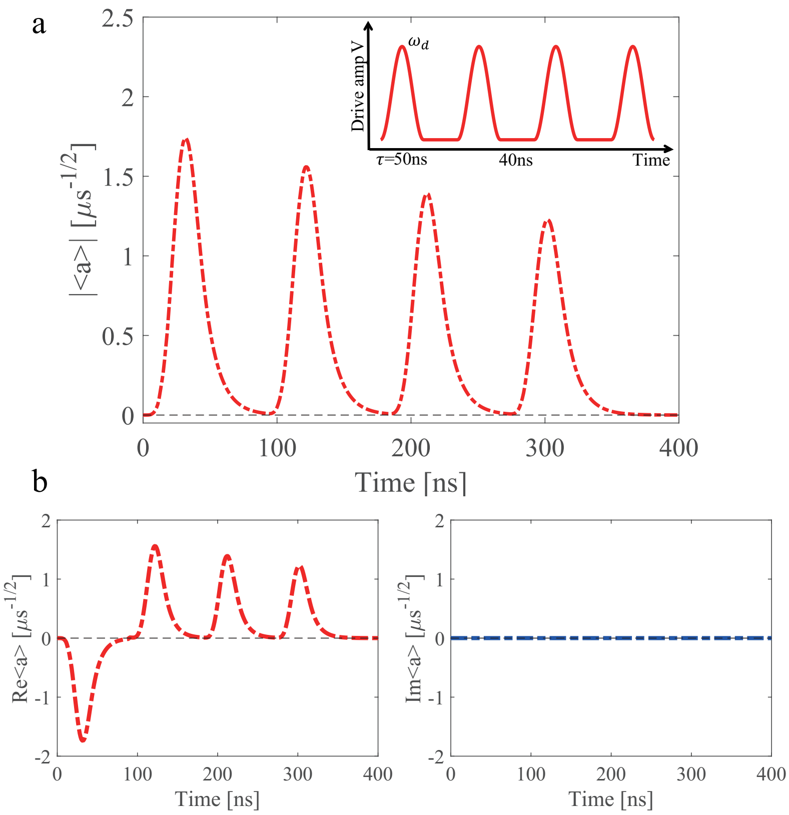

Fig. S2: Generation of four-peaked photon field (a) The amplitude of the four-peaked photon field vs the time using the designed driving pulse in the insert. (b) The real and imag part of the photon field by change the phase of the first peak with π.

In addition, we prepare a multipeaked single photon state by modulating the amplitude and the phase of the driving pulse, which plays an important role in encoding quantum information in time bins. Firstly, we prepare the spin qubit in the state $(|g\rangle + |e\rangle)/\sqrt{2}$, so that the photon field is nonzero and can be characterized. Secondly, as shown in Fig. S2a, the driving pulse on the spin-photon module is separated into four identical independent subpulses with the amplitude $V_0 = 2\pi \times 400$ MHz and the duration $\tau = 50$ ns. The time interval between two neighboring subpulses is 40 ns, in order to minimize the length of the whole pulse and accomplish the generation of multipeaked single photons within the decoherence time. Finally, we simulate the photon field over the time and the results are shown in Fig. S2a.

The single photon state consisting of four peaks corresponds to the shape of the driving microwave pulse. When one photon field is finished, another photon field begins to generate and its peak is smaller than the previous one, since the state $|g0\rangle$ transfers to $|e1\rangle$ under the driving pulse and the population of the state $|g0\rangle$ decreases continuously.

We demonstrate that not only the amplitude but also the phase of the peaks in the photon field can be controlled by adjusting the phase $\phi(t)$ of the driving microwave. As shown in Fig. S2b, we change the phase of one driving subpulse by π, which results in the change of sign in the corresponding photon field while the amplitude of the photon field remains unchanged. The phases of other individual photon peaks can be also modified in the same way. In other words, arbitrary shaped single photons including the amplitude and the phase modulation can be prepared by employing the microwave pulse on the spin-photon module.

Section S7: Integration of dynamical decoupling pulses in the spin-resonator system

Since the fidelity of our scheme depends on coherence time of the spin qubit, we consider the possibility of applying dynamical decoupling pulses in a spin-resonator system, as they dramatically improve coherence time. For a single spin qubit system, dynamical decoupling (DD) pulses can be applied during qubit control and extend the qubit coherence time. Here we can go one step beyond this: we consider a hybrid system that a spin qubit is coupled to a resonator. The targeted manipulation is to transfer a state between the spin qubit and the resonator whose fidelity decays over the task's duration. The goal of mitigating the effect of noisy environment remains, but in this case, care must be taken that the DD pulses should be compatible with the target operation. The main problem is that the DD pulses not only decouple the spin qubit from noise, but also interfere with the spin-resonator coupling that drives the hybrid system to achieve the state transfer. This task can be tackled by inserting a suitably designed operation in periods of a standard DD sequence [11]. In the following, we give a framework for implementation in this hybrid system.

We consider a spin qubit-resonator system governed by a Hamiltonian:

$$H_0 = H_{JC} + H_{DD}, \tag{S21}$$

where $H_{JC} = \frac{1}{2}\Delta\sigma_z + g_{eff}(a^+\sigma_- + a\sigma_+)$ describes a Jaynes-Cummings (JC) like Hamiltonian for interaction between a spin qubit and a resonator with a tunable coupling $g_{eff}(t)$. Note that for clarity, we rewrite H_{mod} in Eq. (S20) as H_{JC} here. Our proposed scheme such as preparation of a shaped single photons, and state transfer between the spin qubit and the resonator rely entirely on manipulation of $g_{eff}(t)$. H_{DD} is a control Hamiltonian for applying DD pulses on the spin qubit.

It is convenient to use the toggling frame description [11], which takes effect of the DD pulses into account:

$$H_i = U^+ H_{JC} U, \tag{S22}$$

where $U = T\exp[-i\int_0^t H_{DD}(t^{'})dt^{'}]$ is the evolution operator of the DD Hamiltonian.

For even number of π-pulses applied, the spin qubit–resonator coupling Hamiltonian H_i preserves the original JC like form. In this duration, the state transfer operation can be implemented. While for odd number of π-pulses, the interaction Hamiltonian H_i is transformed to a different form which leads to unwanted evolution. This interfere can be blocked simply by taking the coupling coefficient $g_{eff}(t) = 0$.

$$\begin{aligned} H_i &= \frac{1}{2}\Delta\sigma_z + g_{eff}(a^+\sigma_- + a\sigma_+), \text{ for even } \pi\text{-pulses} \\ H_i &= -\frac{1}{2}\Delta\sigma_z + g_{eff}(a^+\sigma_+ + a\sigma_-), \text{ for odd } \pi\text{-pulses} \end{aligned} \tag{S23}$$

Following [12], as shown in Fig. S3a, we can design simultaneous modulation of $g_{eff}(t)$ and a sequence of π-pulses. The design includes three elements: (i) applying a DD sequence to the spin qubit to cancel noise effect. (ii) turning on the coupling $g_{eff}(t)$ to transfer a state between the spin qubit and the resonator. (iii) turning off the coupling $g_{eff}(t) = 0$ after each odd-numbered *pi*-pulse to prevent unwanted interfere.

Interlacing a gate operation between DD pulses is not unique. Following [13], we can design another type of implementation as shown in Fig. S3b. An even number of *pi*-pulses is applied to the spin qubit with switching off the coupling $g_{eff}(t) = 0$. Then adjusting the coupling $g_{eff}(t)$ enables state transfer between the spin qubit and the resonator, which is followed by a second DD sequence.

Section S8: Analysis of the spin-photon network

We consider a simple quantum network containing two modules labeled as A and B, shown in Fig. 1a of the main text. The Hamiltonian of the independent module can be described as Eq. (S20). The two nodes is connected with

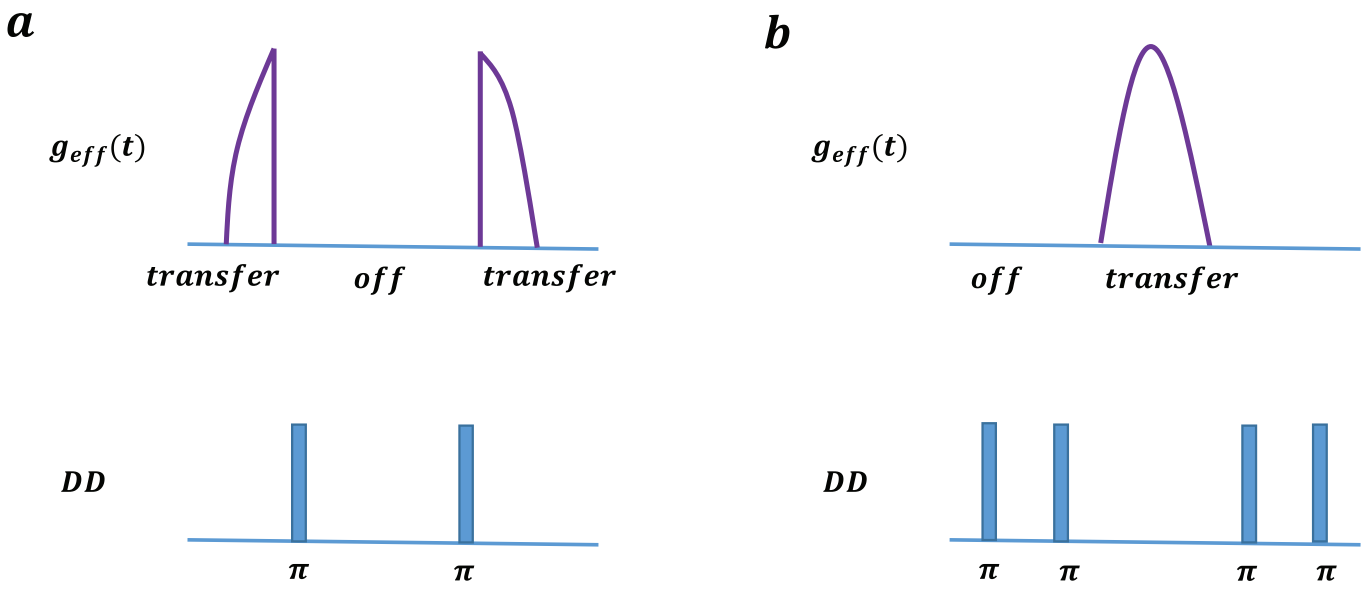

Fig. S3: Combination of spin-resonator operations with dynamical decoupling pluses Protecting the hybrid spin-resonator system from the environmental noise while simultaneously driving state transfer operations can be achieved by using a standard DD sequence and adjusting the spin-resonator coupling $g_{eff}(t)$. Two special implementation examples are illustrated in (a) and (b).

a coaxial line and the Hamiltonian of the network system is given as:

$$\begin{aligned} H_{net} = & \sum_{i=A,B} \{(\hbar g^i_{eff}(t) \left|g0\right\rangle_{ii} \left\langle e1\right| + H.c.) + H_i^{'}\} \\ & + i\hbar \frac{\sqrt{\kappa^A \kappa^B \eta_t}}{2} (\hat{a}_A \hat{a}_B^\dagger - \hat{a}_A^\dagger \hat{a}_B), \end{aligned} \tag{S24}$$

where the subscript $i = A, B$ denotes node A and node B, respectively. We note that the term in the second line of Eq. (S24) represents the process that the sending node is connected to the receiving node with the channel. Here $\kappa^A(\kappa^B)$ is the photon emission rate from the node A(B) and η_t is the photon transmission efficiency in the channel. Then, we put the above Hamiltonian into the master equation and numerically simulate the state transfer across different nodes:

$$\begin{aligned} \dot{\rho} = & -\frac{i}{\hbar}[H_{net}, \rho] \\ & + \sqrt{\eta_t} D[\sqrt{\kappa^A}\hat{a}_A + \sqrt{\kappa^B}\hat{a}_B]\rho \\ & + \sqrt{(1-\eta_t)} D[\sqrt{\kappa^A}\hat{a}_A + \sqrt{\kappa^B}\hat{a}_B]\rho \\ & + \sum_{i=A,B} \{\kappa^i_{int} D[\hat{a}_i]\rho + \gamma^i_l D[\sigma^i_-]\rho + \gamma^i_\varphi D[\sigma^i_z]\rho\} \end{aligned} \tag{S24}$$

where D is the superoperators defined by $D[L]\rho = L\rho L^\dagger - \frac{1}{2}(L^\dagger L\rho + \rho L^\dagger L)$ describing the influence of the environment process on the resonator and the spin qubit. κ_{int} is the internal decay rate of the resonator about the order of 0.1 MHz and can be neglected compared to the photon emission rate of the resonator. $\gamma_1 = 1/T_1$ is the relaxation rate and $\gamma_\varphi = 1/T_\varphi - 1/2T_1$ is the dephasing rate of the spin qubit. The last term in the Hamiltonian and the terms in the second and third line of the master equation, display the process that the output of the emitter node is cascaded to the input of the receiver node through the channel with efficiency η_t [14, 15].

Furthermore, we study the influence of the spin qubit decoherence on the fidelity of the remote entanglement using the master equation. As shown in Fig. S4a, by ranging the spin qubit relaxation time T_1 from 500 ns to 25 μs, and the dephasing time T_φ from 100 ns to 4 μs, we find that longer decoherence time leads to a higher fidelity. On the other hand, the photon transmission efficiency η_t is another factor to limit the remotely entangled state fidelity as shown in Fig. S4b. It is not difficult to find the fidelity increases rapidly as η_t, T_1, T_φ increases and could be increased

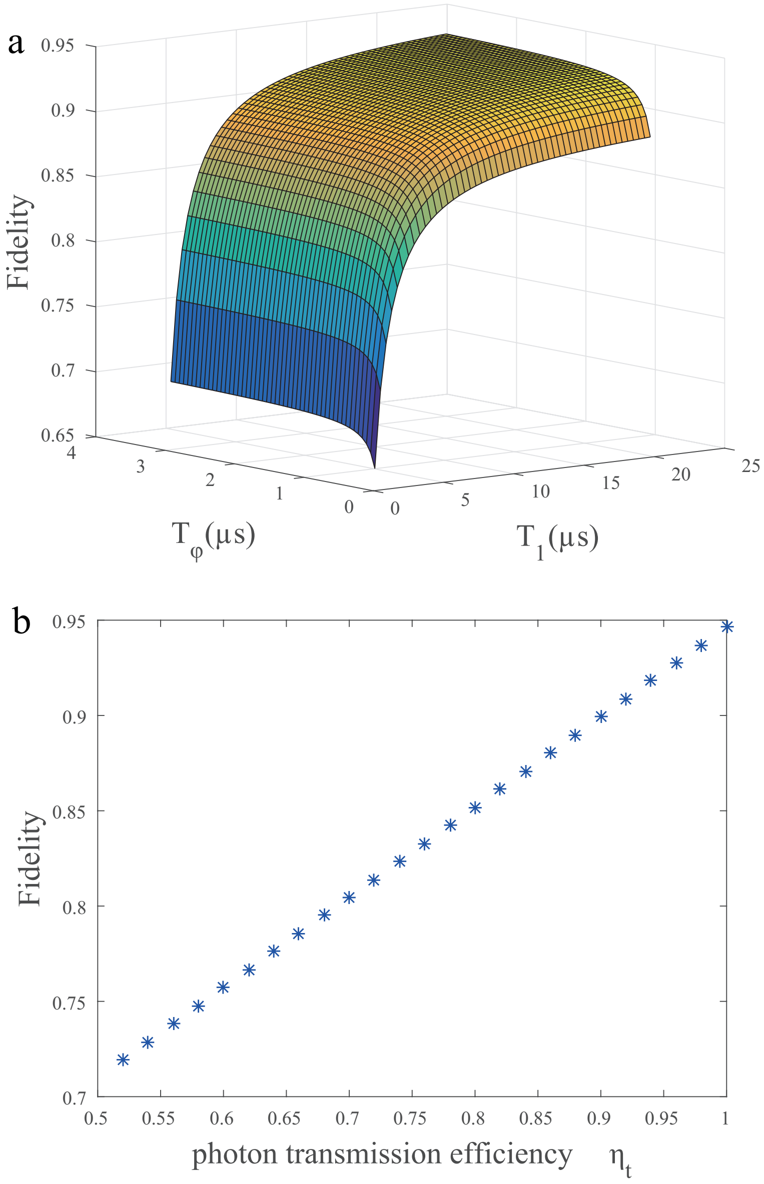

Fig. S4: The fidelity of remote entanglement process as a function of the qubit decoherence and photon loss (a) The fidelity of the two-node entangled state vs the qubit relaxation time T_1 and the dephasing time T_φ. In this case we set the photon transmission efficiency $\eta_t = 92\%$. (b) The fidelity of two-node entangled state vs the photon transmission efficiency η_t through the channel. In this case we set the relaxation and dephasing time of the spin qubit as $T_1 = 10\ \mu$s and $T_\varphi = 1\ \mu$s.

to reach 93% within the existing technology.

Section S9: Performance evaluation of the network architecture

Designing an architecture is an elegant and practical approach for representing and processing quantum information in a scalable quantum computer. There have been many variants of architectures for quantum computation [16–20], each with their own set of pros and cons. We find it convenient to split the architectures into two classes. One class includes the monolithic architecture [21, 22], which incorporates a large array of qubits with neighbours directly connected. The second class contains the network architecture [23–25], in which different nodes are coupled by creating entanglement via a photonic link. The later approach will benefit from using the small simple modules frequently to build large complex systems robustly. The spin-photon module proposed here thus constitutes a kind of building blocks for the network architecture and all higher functions are built upon it.

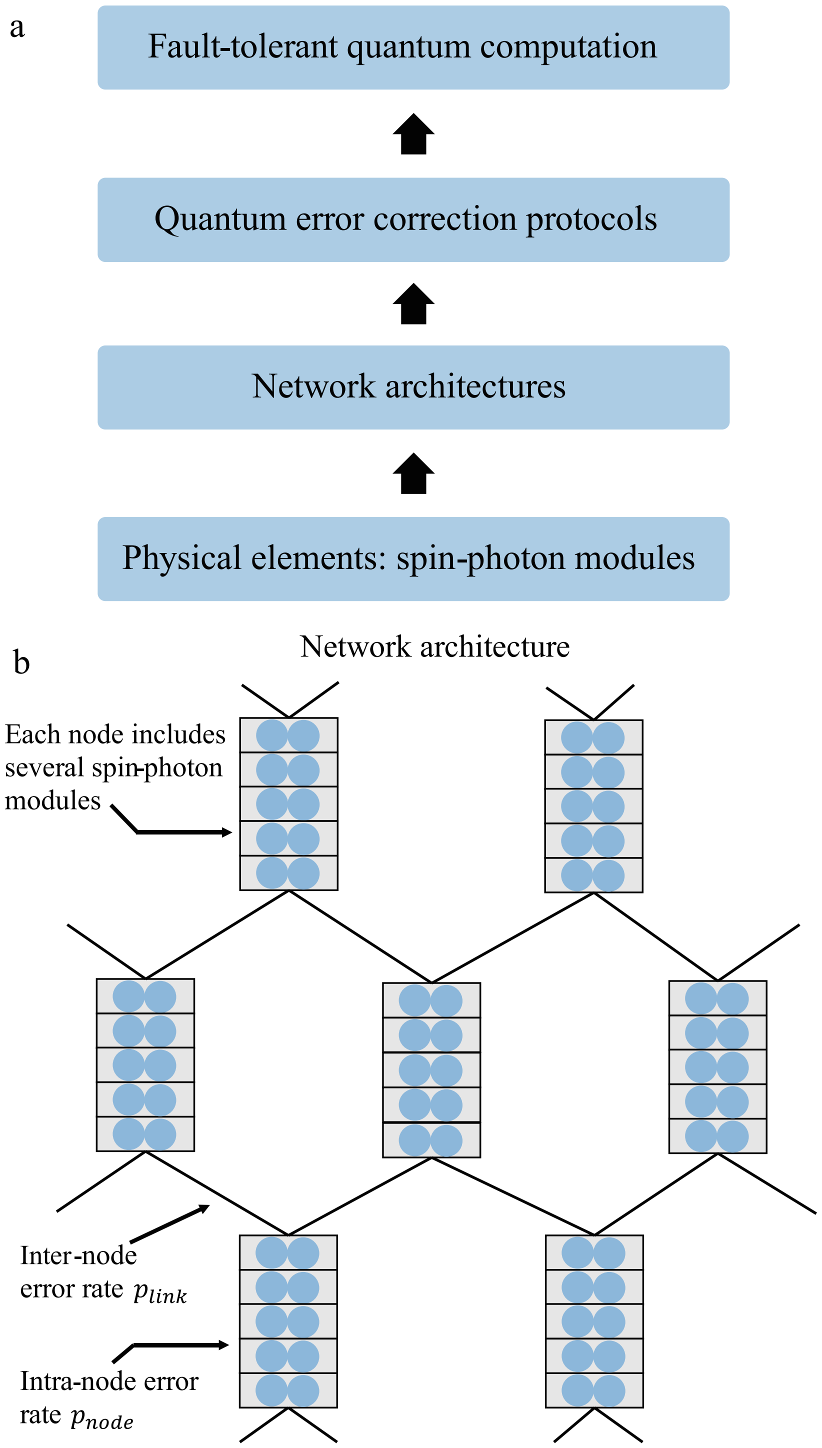

Fig. S5: Network architecture towards scalable quantum computation (a) A view of layered framework for quantum information processing. [16, 18–20], The network architecture can support different quantum error correction protocols if the error rates are below the corresponding threshold. (b) In the network picture, the system is divided up into small nodes, each of which contains a modest number of qubits. The local operations within the node have a error rate p_{node}, while the connections between the nodes have a error rate p_{link}.

	p^{th}_{node}	p^{th}_{link}
NN protocol [23]	0.86%	10%
basic protocol [24]	0.1%	7.7%
medium protocol [24]	0.1%	13.3%
refined protocol [24]	0.1%	19.4%
our architecture	1%	9.2%

Table SI: The threshold of tolerable error rates for intra- and inter-node operations in a network architecture using different error-correction protocols.

However, in a real device performing various operations such as initialising, manipulation and measurement will introduce errors. If the error rates are sufficiently below some threshold, the encoded information can be protected by quantum error correction. As illustrated in Fig. S5a, the act of architecture is to integrate quantum error correction protocols with feasible experimental technology, to find a path towards quantum information processing in a fault-tolerant manner. The threshold of error rate depends on the each particular architecture encoding and error correction protocol.

The focus of the presented work is the design of spin-photon module as a building block for the network architecture, and we envision that many different error correction protocols can be implemented based on our design. There are several proposals for implementing quantum error correction in network architectures. These include 3D topological code in a module architecture [25, 26], and entanglement purification in a distributed architecture [27, 28]. The purification idea is using the intra-node operations to remove errors in noisy inter-node links. There are a number of works to extend this idea with a more sophisticated purification protocols [23, 24, 29–31]. Here we employ such techniques [23, 24] in order to estimate the practical error rate threshold.

As shown in Fig. S5b, small nodes are connected with photonic links in a network architecture, where each nodes contains a number of data qubits and ancilla qubits. The protocols in [23, 24] are to first prepare a shared entangled state between the nodes involved and then purify the raw entanglement using the intra-node operations. The purified entangled state can be used as a resource to error correction in this architecture. Fig. S4b defines two error rates: one is p_{node} due to the failures in initialising, manipulation and measurement inside a node, the other is p_{link} for errors in entanglement preparations across different nodes. The extensive analyses have indicated the thresholds for a node's own internal error rate and inter-node error rate in Table SI.

The threshold of inter-node error rate p^{th}_{node} is somewhat low, of the 1% order. Such as demand is not prohibitive, since this level of fidelity is already achievable using microwave pulses in laboratory quantum dot systems [32–37]. Actually, we find the single-qubit operations using microwave pulse can reach very high fidelity as 99.9% in the numerical simulations of the spin-photon module. On the other hand, for a NN protocol which is compatible with recent experiments [23], the threshold of inter-node error rate p^{th}_{link} is of 10%. In particular, we find that our spin-photon network can succeed provided that the error rate for the entanglement over a remote link is as $p_{link} \approx 9.2\%$. Thus our architecture achieves the error rates for the intra-node and inter-node operations which are approaching or below the fault-tolerable thresholds.

The proposed spin-photon network is also amenable to other error correction protocols such as the basic protocol [24], which has lower threshold but a more resources saving. We remark that according to the calculations in section S7, the error rate for remote entanglement can be reduced to 7%. This is particular promising in this network architecture, given the ability to inter-node operations with high fidelity.

[1] Foletti, S. et al. Universal quantum control of two-electron spin quantum bits using dynamic nuclear polarization. *Nat. Phys.* **5**, 12 (2009).

[2] Pioro-Ladriere, M. et al. Electrically driven single-electron spin resonance in a slanting Zeeman field. *Nat. Phys.* **4**, 10 (2008).

[3] Wu, X. et al. Two-axis control of a singlet-triplet qubit with an integrated micromagnet. *Proceedings of the National Academy of Sciences* **111**, 33 (2014).

[4] Taylor, J. M. et al. Relaxation, dephasing, and quantum control of electron spins in double quantum dots. *Phys. Rev. B* **76**, 3 (2007).

[5] Jin, P.-Q. et al. Strong coupling of spin qubits to a transmission line resonator. *Phys. Rev. Lett.* **108**, 19 (2012).

[6] Petta, J. R. et al. Coherent manipulation of coupled electron spins in semiconductor quantum dots. *Science* **309**, 5744 (2005).

[7] Koppens, F. H. L. et al. Driven coherent oscillations of a single electron spin in a quantum dot. *Nature* (London) **442**, 766 (2006).
[8] Nowack, K. C. et al. Coherent control of a single electron spin with electric fields. *Science* **318**, 1430 (2007).
[9] Blais, A. et al. Cavity quantum electrodynamics for superconducting electrical circuits: An architecture for quantum computation. *Phys. Rev. A* **69**, 6 (2004).
[10] James, D. F. Quantum computation with hot and cold ions: an assessment of proposed scheme. *Fortschr. Phys.* **48**, 823 (2000).
[11] Suter, D. & Alvarez, G. A. Protecting quantum information against environmental noise. *Rev. Mod. Phys.* **88**, 041001 (2016).
[12] Beaudoin, F., Blais, A. & Coish, W. A. Hamiltonian engineering for robust quantum state transfer and qubit readout in cavity QED. *New J. Phys.* **19**, 023041 (2017).
[13] Barthel, C., Medford, J., Marcus, C. M., Hanson, M. P., & Gossard, A. C. Interlaced dynamical decoupling and coherent operation of a singlet-triplet qubit. *Phys. Rev. Lett.* **105**, 266808 (2010).
[14] Gardiner, C. & Zoller, P. Quantum noise: a handbook of Markovian and non-Markovian quantum stochastic methods with applications to quantum optics, volume 56. Springer Science & Business Media, (2004).
[15] Kurpiers, P. et al. Deterministic quantum state transfer and remote entanglement using microwave photons. *Nature* **558**, 264–267 (2018).
[16] Devoret, M. H., & Schoelkopf, R. J. Superconducting circuits for quantum information: an outlook. *Science* **339**, 1169-1174 (2013).
[17] Monroe, C., & Kim, J. Scaling the ion trap quantum processor. *Science* **339**, 1164-1169 (2013).
[18] Jones, N. C. et al. Layered architecture for quantum computing, *Phys. Rev. X* **2**, 031007 (2012).
[19] Gambetta, J. M., Chow, J. M., & Steffen, M. Building logical qubits in a superconducting quantum computing system. *npj Quantum Information* 3: 2 (2017).
[20] Wehner, S., Elkouss, D., & Hanson, R. Quantum internet: A vision for the road ahead. *Science* **362**, eaam9288 (2018).
[21] Fowler, A. G., Mariantoni, M., Martinis, J. M., & Cleland, A. N. Surface codes: Towards practical large-scale quantum computation. *Phys. Rev. A* **86**, 032324 (2012).
[22] Ghosh, J., Fowler, A. G., & Geller, M. R. Surface code with decoherence: An analysis of three superconducting architectures. *Phys. Rev. A* **86**, 062318 (2012).
[23] Nickerson, N. H., Li, Y., & Benjamin, S. C. Topological quantum computing with a very noisy network and local error rates apprroaching one percent. *Nat. Commun.* **4**, 1756 (2013).
[24] Nickerson, N. H., Fitzsimons, J. F., & Benjamin, S. C. Freely scalable quantum technologies using cells of 5-to-50 qubits with very lossy and noisy photonic links. *Phys. Rev. X* **4**, 041041 (2014).
[25] Nemoto, K. et al. Photonic architecture for scalable quantum information processing in diamond. *Phys. Rev. X* **4**, 031022 (2014).
[26] Monroe, C. et al. Large scale modular quantum computer architecture with atomic memory and photonic interconnects. *Phys. Rev. A* **89**, 022317 (2014).
[27] Dur, W., & Briegel, H.-J. Entanglement Purification for Quantum Computation. *Phys. Rev. Lett.* **90**, 067901 (2003).
[28] Jiang, L. et al. Distributed quantum computation based on small quantum registers. *Phys. Rev. A* **76**, 062323 (2007).
[29] Campbell, E. T. Distributed quantum-information processing with minimal local resources, *Phys. Rev. A* **76**, 040302 (2007).
[30] Fujii, K., Yamamoto, T., Koashi, M., & Imoto, N. A. Distributed architecture for scalable quantum computation with realistically noisy devices, arXiv:1202.6588v1.
[31] Zwerger, M., Briegel, H. J., & Dur, W. Hybrid architecture for encoded measurement-based quantum computation, *Sci. Rep.* **4**, 5364 (2014).
[32] Veldhorst, M. et al. An addressable quantum dot qubit with fault-tolerant control-fidelity. *Nat. Nanotechnol.* **9**, 981–985 (2014).
[33] Yoneda, J. et al. A quantum-dot spin qubit with coherence limited by charge noise and fidelity higher than 99.9%. *Nat. Nanotechnol.* **13**, 102-106 (2018).
[34] Yang, C. H. et al. Silicon qubit fidelities approaching incoherent noise limits via pulse optimisation. *Nat. Electronics.* **2**, 151 (2019).
[35] Huang, W. et al. Fidelity benchmarks for two-qubit gates in silicon. *Nature* **569**, 532–536 (2019).
[36] Nichol, J. M. et al. High-fidelity entangling gate for double-quantum-dot spin qubits. *npj Quant. Inf.* **3**, 3 (2017).
[37] Cerfontaine, P. et al. Closed-loop control of a GaAs-based singlet-triplet spin qubit with 99.5% gate fidelity and low leakage. arXiv:1906.06169.
